# NIR-II Fluorescent Probes for Fluorescence-Imaging-Guided Tumor Surgery

**DOI:** 10.3390/bios14060282

**Published:** 2024-05-30

**Authors:** Zia Ullah, Shubham Roy, Jingshi Gu, Sai Ko Soe, Jian Jin, Bing Guo

**Affiliations:** 1 School of Science, Shenzhen Key Laboratory of Flexible Printed Electronics Technology, Shenzhen Key Laboratory of Advanced Functional Carbon Materials Research and Comprehensive Application, Harbin Institute of Technology, Shenzhen 518055, China; ziadrishak@gmail.com (Z.U.); shubham@hit.edu.cn (S.R.); kaunggyi711@gmail.com (S.K.S.); 2 Education Center of Experiments and Innovations, Harbin Institute of Technology, Shenzhen 518055, China; gujingsi@hit.edu.cn

**Keywords:** fluorescence imaging, NIR-II fluorescence imaging, image-guided tumor surgery, fluorescent probes, organic and inorganic fluorescent probes

## Abstract

Second near-infrared (NIR-II) fluorescence imaging is the most advanced imaging fidelity method with extraordinary penetration depth, signal-to-background ratio, biocompatibility, and targeting ability. It is currently booming in the medical realm to diagnose tumors and is being widely applied for fluorescence-imaging-guided tumor surgery. To efficiently execute this modern imaging modality, scientists have designed various probes capable of showing fluorescence in the NIR-II window. Here, we update the state-of-the-art NIR-II fluorescent probes in the most recent literature, including indocyanine green, NIR-II emissive cyanine dyes, BODIPY probes, aggregation-induced emission fluorophores, conjugated polymers, donor–acceptor–donor dyes, carbon nanotubes, and quantum dots for imaging-guided tumor surgery. Furthermore, we point out that the new materials with fluorescence in NIR-III and higher wavelength range to further optimize the imaging results in the medical realm are a new challenge for the scientific world. In general, we hope this review will serve as a handbook for researchers and students who have an interest in developing and applying fluorescent probes for NIR-II fluorescence-imaging-guided surgery and that it will expedite the clinical translation of the probes from bench to bedside.

## 1. Introduction

Tumor surgery without visual navigation has been the most challenging task to accomplish in the past few years [1]. Scientists have devised various imaging modalities to solve this problem for precise diagnosis and removal of the tumor during the surgical process [2]. In the realm of medical diagnostics, professionals commonly use X-ray imaging, CT, MRI, PET, and endoscopy [3]. All these imaging modalities are very expensive and contain harmful radiations which are dangerous for the patients and may cause serious complications [4,5,6].

Fluorescence imaging (FI) is a cutting-edge imaging fidelity modality for cancer imaging and in vivo visualization of tumors. In medicine, FI aids in cancer detection, tissue visualization during surgeries, and drug deliveries. FI is categorized as visible (400–700 nm), first near-infrared (NIR-I, 700–900), and second near-infrared (NIR-II, 1000–1700 nm) depending on the fluorescence window of the fluorescent material [7]. Currently, scientists are working on the NIR-II FI because of its several advantages over the other two [8]. Commonly cited estimates suggest penetration depths of NIR-II radiations from a few millimeters to several centimeters, depending on tissue type and experimental conditions [9]. In 2016, Shao et al. reported in one of their experiments that NIR-II emissive fluorescence can be used to clearly visualize at a tissue depth of 9 mm and capture optical signals at a tissue depth of 23 mm [10]. Hu et al., 2020, reported penetration depths of approximately 4 to 5 cm in mice using NIR-II FI [6]. NIR-II FI has a greater signal-to-background ratio (SBR), higher biocompatibility reduced autofluorescence, spatial resolution, and excellent targeting properties [11,12]. All these properties make NIR-II fluorophores invaluable for applications like imaging-guided surgery (IGS) of cancer, cancer diagnosis, drug delivery monitoring [13,14] and studying cellular processes with exceptional precision [15].

The endogenous bodily proteins and lipids have no absorption in the NIR-II window as compared to the visible and NIR-I spectral ranges. This property of NIR-II radiation can reduce auto-fluorescence, light scattering, and background noise, providing higher SBR with greater sensitivity. The enhanced spatial resolution and deeper penetration depth of NIR-II FI are attributed to its longer wavelength possessing minimal invasiveness. Moreover, NIR-II fluorophores exhibit excellent targeting properties. Researchers can conjugate NIR-II fluorophores to molecular probes or antibodies that selectively bind to a target of interest, such as tumor cells or specific proteins. When introduced into biological systems, these targeted NIR-II probes provide real-time, high-contrast imaging, facilitating the visualization and tracking of specific biomarkers or pathological features. However, water has strong absorption in the NIR-II region, but the absorption peak is very sharp, with minimal autofluorescence and background noise.

However, the NIR-II window possesses higher spatial resolution and deeper penetration depth as compared to the visible and NIR-I spectral ranges, but the results can be compromised by the absorption region of water. Moreover, tissue autofluorescence, background noise, and damage to the biological tissues are also issues still to be addressed. The spatial resolution and penetration depth can be further enhanced by using a third near-infrared (NIR-III, 1700–2500 nm) window. Because of the longer wavelength and lower energy, it possesses maximum spatial resolution and deeper penetration depth, with minimal side effects. 

**Figure 1:**
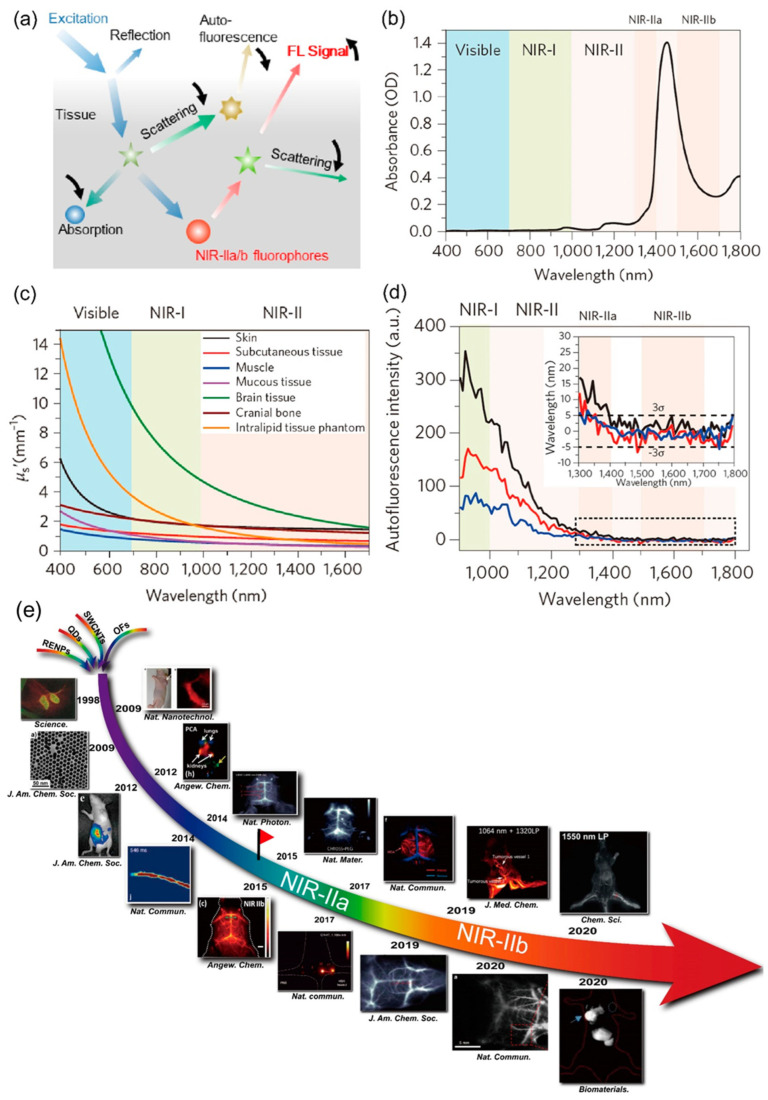
(**a**) Demonstration of how light interacts with living tissue in fluorescence imaging [16] (Reproduced with permission from Copyrights 2022, AMERICAN CHEMICAL SOCIETY Publications). (**b**) Absorption spectra of water for a path length of 1 mm. (**c**) Reduced scattering coefficient of various bodily tissues and a distorting intralipid tissue mimic across wavelengths spanning 400–1700 nm. (**d**) Autofluorescence profiles of ex vivo mice heart tissues (blue), spleen (red), and mouse liver (black) when excited by 808 nm, displaying minimal autofluorescence within the 1500 nm NIR-IIb range [17] (Panels (**b**–**d**) are reproduced with permission from Copyrights 2017, NATURE). (**e**) Chronology detailing the advancement of NIR-IIa/IIb imaging [16] (Reproduced with permission from Copyrights 2022, AMERICAN CHEMICAL SOCIETY Publications).

Liu et al., 2021, offered a comprehensive report concerning the development, characteristics, molecular fluorescence imaging, theranostics of inorganic and organic NIR fluorophores within the NIR-IIa/IIb range, and design principles of functional NIR-IIa/IIb biomaterials (Figure 1) [16,17]. Zhu et al., 2019, discussed the clinical applications of NIR-II imaging, the production and molecular compositions, chemical and optical characteristics, the process of attachment to biological molecules, behavior in biological systems, the employment of NIR-II dyes for cancer diagnosis and surgical guidance through whole-body imaging, and explored their application in NIR-II FI [18]. Li et al., 2021, outlined the fundamental design, and operational principles of NIR-II FI in guiding surgery through NIR-I/II imaging [19]. Zhang et al., 2021, provided an overview of NIR-II fluorescence agents and their diverse applications in guiding tumor surgeries [20]. Zhu et al., 2023, offered an overview of the mechanism of interaction, improved performance, capability of tumor targeting, and in vivo fluorescence imaging of the NIR-II fluorophores [21]. Wang et al., 2023, summarized the versatile designs and functionality and applications of NIR-II fluorescent probes for in vivo processes in multichannel biosensing, biological process monitoring, cellular tracking, and pathological analysis [22]. Chen et al., 2023, summarized different strategies to optimize NIR-II fluorescent probes, traditional chemical modification, modern bioengineering processes, and production of NIR-II fluorescent probes with the help of endogenous serum protein and exogenous gene-editing proteins [23].

**Scheme 1:**
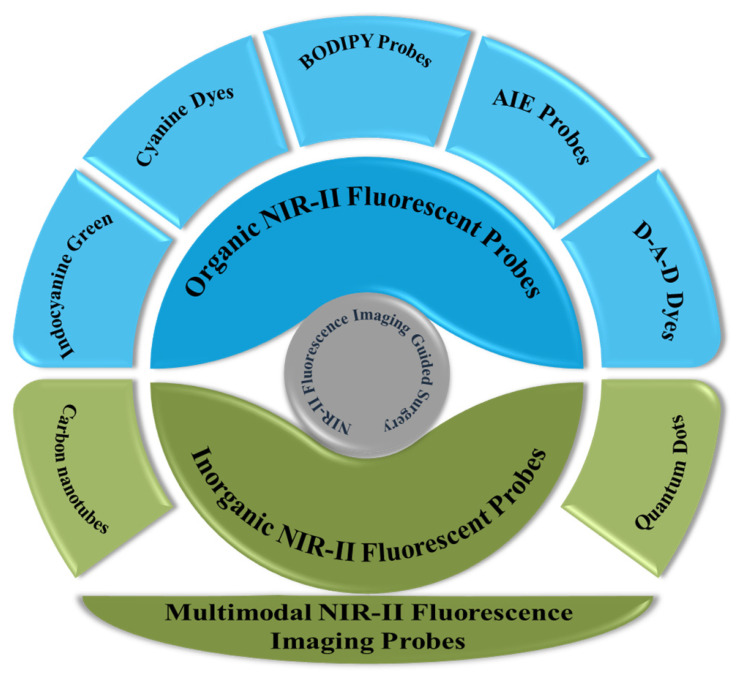
Schematic demonstration of the state-of-the-art NIR-II fluorescent materials used for imaging-guided tumor surgery in the most recent literature.

Most of these review papers discuss the probes demonstrating NIR-II fluorescence in biomedical FI and biosensing in broader terms. More importantly, work on NIR-II fluorescence imaging-guided surgery (IGS) is booming in the medical realm. In conclusion, NIR-II FI is a rapidly advancing field with a strong rationale based on its unique properties and capabilities. As technology continues to evolve, NIR-II imaging is poised to play an increasingly important role in advancing our knowledge of biology and improving healthcare outcomes [24]. Therefore, there is a need to offer a timely review that clearly highlights the applications of NIR-II fluorescent materials in imaging-guided tumor surgery. Generally, this review covers the applications and desired features of organic and inorganic NIR-II fluorescent materials in imaging-guided tumor surgery (Scheme 1). Moreover, multimodal NIR-II FI for IGS and the prospective application of NIR-III FI for enhanced spatial resolution and penetration depth are also summarized.

## 2. NIR-II Fluorescent Probes for IGS

Fluorescent materials, when irradiated by a specific light source, show fluorescence in the NIR-II window and are termed NIR-II fluorescent probes. In this section, we are going to discuss the organic and inorganic NIR-II fluorescent materials used in IGS.

### 2.1. Organic Probes

NIR-II fluorescent probes containing organic fluorophore are termed organic fluorescent probes. In this section, we are going to discuss the organic NIR-II fluorescent probes that have been used for imaging-guided tumor surgery.

#### 2.1.1. Indocyanine Green: Food and Drug Administration (FDA)-Approved Probe

Indocyanine green (ICG), an FDA-approved fluorescent dye initially used for IGS in the NIR-I region, gained newfound significance in biomedical imaging due to its emergence in the NIR-II region with an excitation wavelength at 789 nm. This particular probe boasted rapid liver clearance within 10 h, maintaining a stable SBR of up to 11.5. Leveraging this advancement, this novel NIR-II agent proved adept at delineating tumor margins and aiding in surgery [6,25,26].

Wang et al., 2019, showcased the potential of ICG in NIR-II emission for swift translation into clinical bioimaging. To make ICG viable, a complex with bovine serum albumin was created, loaded into red blood cells, and then further modified into a red-blood-cell-based probe (RBCp). RBCp exhibited improved tumor retention kinetics, and it was revealed in experiments in liver-adenocarcinoma-bearing mice that RBCp exhibited clear NIR-II fluorescence signals after 2 h and minimized at around 10 h, showcasing the metabolism of RBCp at tumor sites. The SBR remained stable at ~11.5 from 10 to 13 h post-injection, providing a crucial “surgical window” for precise IGS of the tumor [27,28].

**Figure 2:**
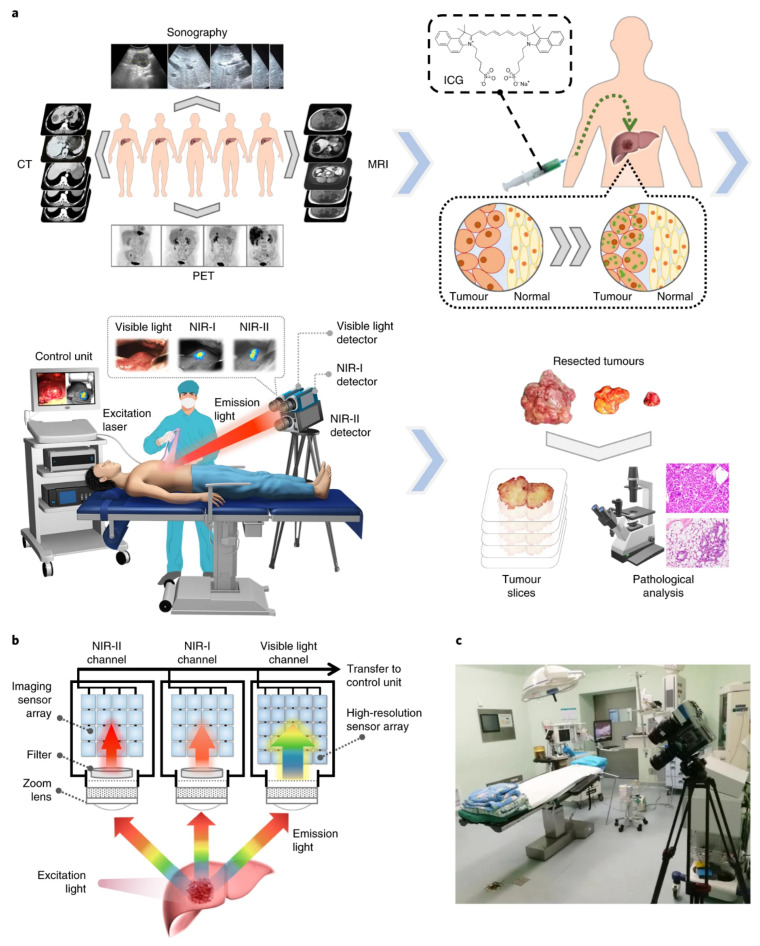
(**a**) Individuals diagnosed with liver cancer were investigated in this research and underwent preoperative diagnostic imaging, which included computed tomography, magnetic resonance imaging, ultrasonography, and positron emission tomography scans. Patients were intravenously injected with ICG for routine preoperative liver function assessment prior to surgery. Between 1 and 7 days later, patients underwent laparotomy. The surface of the liver was examined using an integrated multispectral imaging device, capturing all visible and NIR-I/II images. Tumors were identified and removed with the assistance of ultrasonography and NIR-I FI. Postoperatively, all the visible and NIR-I/II images of the removed tumor were obtained, followed by pathological investigation of the removed tumors. (**b**) Diagram illustrating the integrated multispectral FI device. (**c**) The personalized multispectral FI instrument positioned within the operation theater [6] (Reproduced with permission from Copyrights 2019, NATURE).

Hu et al., 2020, introduced an optical imaging device utilizing the dye ICG to assist in the fluorescence imaging-guided surgical removal of primary and metastatic liver tumors in 23 patients. Their study revealed that intraoperative NIR-II imaging offered superior sensitivity, higher SBR, and an increased rate of tumor detection compared to NIR-I imaging. They suggested that the integration of NIR-I and NIR-II spectral ranges and appropriate fluorescence probes could enhance IGS in clinical settings (Figure 2) [6].

Although ICG is an FDA-approved fluorescent probe, it has several limitations necessary to be addressed to further improve surgical outcomes. ICG lacks the tumor-targeting ability, which is very important to further optimize the efficiency of the fluorescent probes. Moreover, ICG is also not an activatable fluorescent probe for effective tumor diagnosis.

#### 2.1.2. Cyanine Dyes

Another realm of investigation involves the modification of cyanine dyes to change their emission towards the NIR-II wavelength, complementing the utilization of off-peak-tail emission from NIR-I cyanine dyes [29]. However, extending the polymethine chain can compromise stability and fluorescence quantum yield (QY). Therefore, an alternative strategy has emerged, involving alterations in the terminal heterocyclic groups. This approach has led to the commercial production of certain polymethine dyes, including IR-26, IR-1048, and IR-1061. Building upon these early achievements, scientists have endeavored to develop innovative NIR-II cyanine dyes with enhanced biocompatibility and fluorescence QY [30]. To enhance the fluorescence QY, Zhu et al. replaced the S atom in IR-26 with an O atom. To counter the resulting blue shift in oxygen absorption, electron-donating dimethylamino groups were incorporated. This led to the creation of the flavylium polymethine fluorophore Flav7, which boasts a QY of 0.53 with absorptive and emissive peaks extending outside 1000 nm [31]. In a subsequent exploration, Wang et al. investigated the heptamethine chain of IR-26 and transitioned to a smaller pentamethine chain. Electron-donating diethylamino units were introduced for bathochromic adjustments. The resultant dye, BTC1070, with its abbreviated polymethine chain, demonstrated enhanced photostability in aqueous solutions and significantly reduced solvatochromic quenching when compared to IR-26. These improvements allowed BTC1070 to achieve peak absorption/emission above 1000 nm, yielding illumination seven times greater [32,33].

In their 2022 study, Tian et al. carried out IGS targeting SLNs in a metastatic tumor model. SLN metastasis represents a critical initial step in the metastatic process and serves as a primary prognostic indicator for various cancers, including melanoma, breast, cervical, uterine, and lung cancers. To precisely locate SLNs, IR-783@TDIII was injected directly into 4T1 tumor-bearing mice. Within 15 min of administration, the complex provided clear imaging contrast and demonstrated high photostability, facilitating efficient SLN identification. IGS was carried out in a brightly lit environment with laser exposure, enabling accurate localization and precise excision of the SLNs [34].

In 2023, Zhang et al. engineered a cyanine–albumin complex, with its well-established advantages, which emerged as a promising imaging probe with significant clinical potential for generating accurate bodily images. Additionally, cyanine-7 heptamethine dyes, specifically those with meso-Cl and recognized as tumor-targeting dyes, are anticipated to accumulate in solid tumors selectively. Notably, cyanine dyes like 1-Cl−5-Cl exhibit localization in various solid tumors such as lung cancer, prostate cancer, gastric cancer, and kidney while avoiding healthy tissue. The accumulation characteristics of these cyanine dyes with meso-Cl are predominantly attributed to their covalent linkage to albumin in vivo [21]. Moreover, it is hypothesized that the complex could be effectively internalized by tumor cells due to the overexpression of albumin receptors in tumor cells. Building upon this understanding, these tumor-targeting dyes have been employed to confer tumor-targeting capabilities to other imaging agents through covalent conjugation while retaining the integrity of the meso-Cl [35].

#### 2.1.3. BODIPY Probes

Fluorophores derived from dipyrromethene boron difluoride (BODIPY) exhibit outstanding characteristics such as robust photostability, versatility in chemical amendments, a higher molar extinction coefficient, and compatibility with biological systems [36]. Recently, BODIPY/Aza-BODIPY fluorescent materials featuring NIR-II absorption or emission have been developed [37]. Bai et al. engineered a set of D-A-D type fluorescent materials by integrating groups with the potential ability to donate electrons at the 3,5-positions of aza-BODIPY [38]. Incorporating electron donors resulted in the extension of absorption and emission wavelengths towards the red end of the spectrum. As a result, the fluorophores synthesized exhibited peak emission wavelengths at 960, 1030, and 1060 nm.

Activatable nanoprobes are composed of two organic fluorescent mioties: a boron-dipyrromethene fluorescent dye, engineered to emit NIR-II light exclusively in H_2_S presence; and an aza-BODIPY fluorescent dye, which remains unresponsive to H_2_S, acting as a reference. The fabrication of these nanoprobes involves two steps: encapsulating boron-dipyrromethene and aza-BODIPY within the hydrophobic core of self-assembled micellar aggregates; and subsequently employing in situ cross-linking of the shell to produce hydrophilic core–shell silica nanocomposites with a covalently cross-linked silica shell. As a result, the silica cross-linkers establish a protective barrier, high water solubility, exceptional biocompatibility, and rapid responsiveness, ensuring the stable retention of two dyes in the same nanoparticle cavity [39,40].

**Figure 3:**
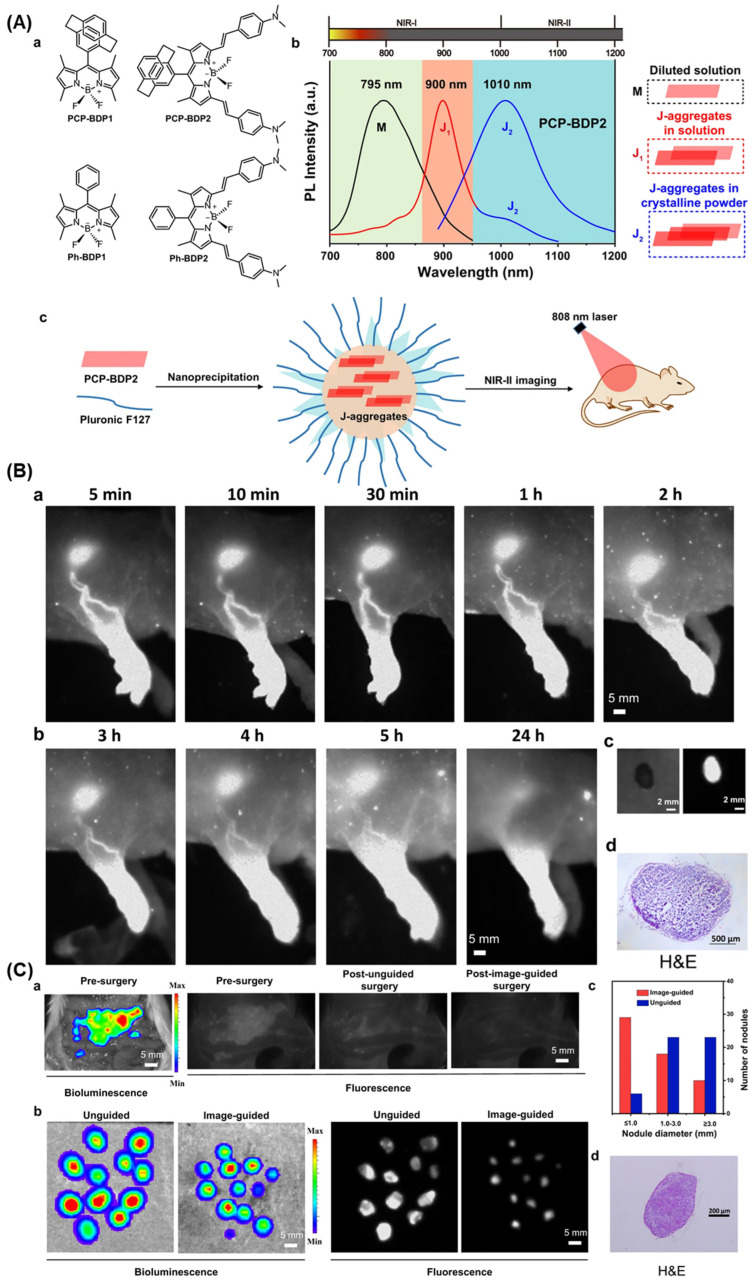
(**A**) (**a**). Molecular structures of different probes. (**b**). Fluorescence emission of sample in dilute solution, THF-H_2_O binary solvents, and in powdered crystalline form. The red parallelogram represents the single molecule of the sample. (**c**). Diagram illustrating the assembly process of sample nanoparticles for NIR-II FI. (**B**) (**a**,**b**). FI of lymph nodes and lymphatic vessels at different time intervals. (**c**). fluorescent images of the sentinel lymph node from the mice. (**d**). H&E staining of the lymph node. (**C**) (**a**). FI of the abdominal cavity before and after the tumor resection. (**b**). NIR-II fluorescence signals of tumor unguided resection of nodules and resection guided by sample nanoparticles. (**c**). Statistical demonstration of nodule size resected from NIR-II fluorescence-guided surgery and unguided resection. (**d**). H&E staining of resected tumor nodules [41] (Reproduced with permission from Copyrights 2021, NATURE).

Liu et al., 2021, prepared meso-[2.2]paracyclophanyl-3,5-bis-N,N-dimethylaminostyrl BODIPY (PCP-BDP2) as an exemplary BODIPY dye with J-aggregation produced NIR-II fluorescence. An emission wavelength at 1010 nm in the J-aggregation state was demonstrated by PCP-BDP2. The mechanistic investigation revealed that the conjugation and the conjugation effect of the PCP group on the BODIPY have a prime role in photophysical properties’ tuning and J-aggregation. Notably, PCP-BDP2 J-aggregates can be applied to the imaging of lymph nodes and fluorescence IGS in nude mice, showing their potential medical application. This study shows BODIPY dye as a substitute J-aggregation mioty for engineering NIR-II fluorescent materials (Figure 3) [41].

#### 2.1.4. AIE Probes

Organic nanoparticles often have low QY due to the arrangement of fluorescent molecules within them, which can cause “aggregation-caused quenching” (ACQ). However, some fluorescent compounds exhibit the opposite phenomenon, known as aggregation-induced emission (AIE). These AIE luminous (AIEgens) become brighter when in solid or aggregated forms, such as within nanoparticles. Encapsulating AIEgens in nanoparticles can create “AIE nanoparticles”, which have higher fluorescence by increasing the encapsulated AIEgens. This unique property gives AIE nanoparticles a relatively high QY and fluorescence intensity, suitable for in vivo imaging. Using known AIEgens with a D-A-D structure, researchers replaced S with a Se atom in the acceptor unit, resulting in a novel AIEgen called BPST. This new AIEgen displayed shifted absorption and emission wavelength compared to the parent molecules. After incorporating BPST molecules into nanoparticles (L897 nanoparticles), the resulting nanoparticles emitted light at 897 nm with an extended tail up to 1200 nm and a QY of 5.8%. Testing these L897 nanoparticles in mice, researchers successfully demonstrated clear NIR-II FI of blood vessels, lymphatic vessels, and tumors [42].

**Figure 4:**
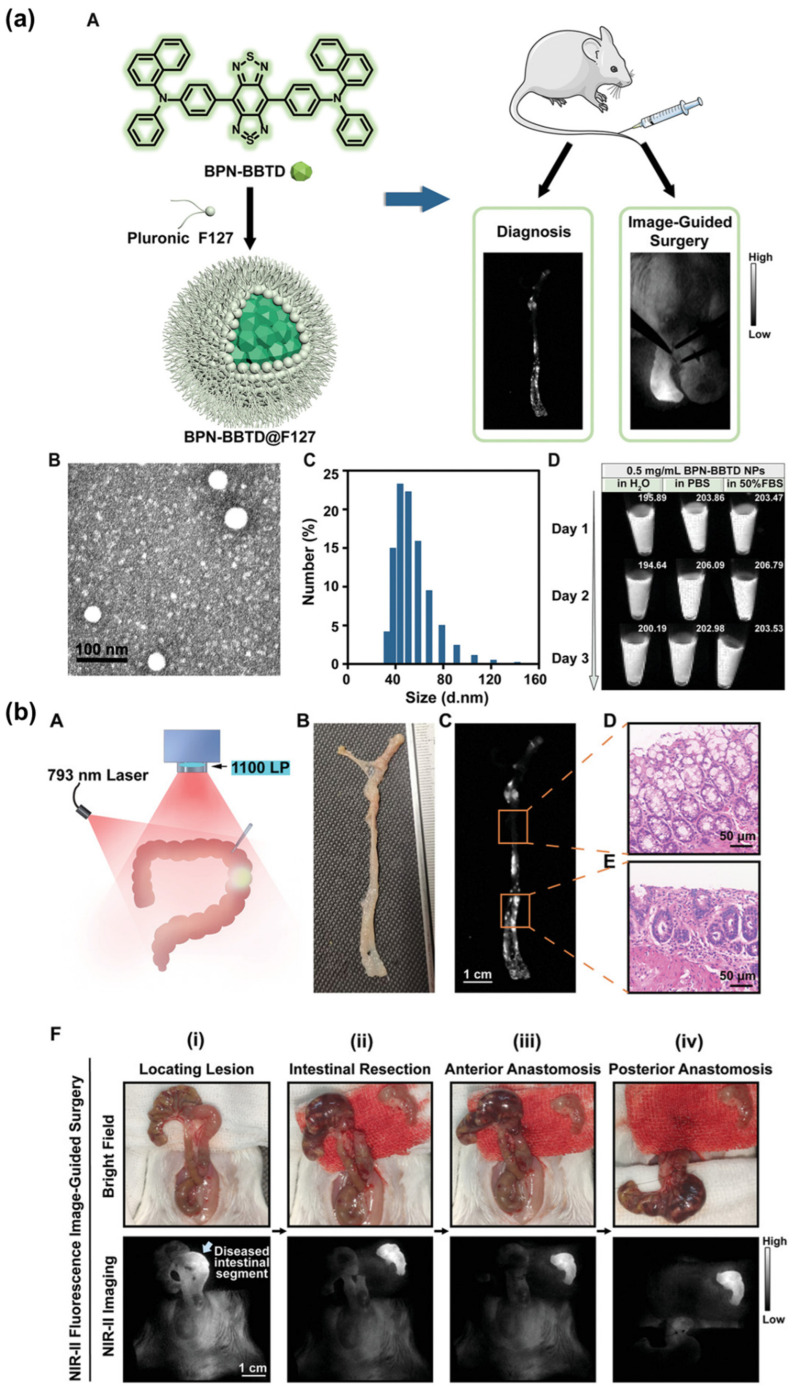
(**a**) Synthesis and characterization of sample nanoparticles: (**A**) demonstration of the manufacturing process and potential uses of sample nanoparticles. (**B**) A single TEM picture showcasing sample nanoparticles. (**C**) DLS assessment displaying the diameter of sample nanoparticles. (**D**) NIR-II images and the collective fluorescence intensities of sample nanoparticles in different solvents over 3 days. (**b**) NIR-II FI was employed for intraoperative guidance of bowel segments exhibiting significant inflammation. (**A**) Illustration depicting the NIR-II fluorescence IGS. (**B**) Macroscopic appearance of the intestinal section in mice with colitis. (**C**) NIR-II imaging capturing the intestinal segment in mice with colitis. (**D**,**E**) H&E staining of the intestinal segment with minimal NIR-II fluorescence and the segment displaying noticeable NIR-II fluorescence. (**F**) The precisely identified intestinal segment demonstrating intense NIR-II fluorescence when excited with a 793 nm laser [44] (Reproduced with permission from Copyrights 2021, WILEY).

Fan et al. engineered an AIE fluorescent material, termed BPN-BBTD, which emits NIR-II light strongly, applying it to visualize blood vessels and tumors. The donor–acceptor formation grants powerful NIR-II fluorescence characteristics to BPN-BBTD, boasting QY by 1.8%. BPN-BBTD was further enclosed within Pluronic F127 to form nanoparticles for expanded biomedical use. Spectral analysis indicated an absorption λ_max_ at ≈710 nm and an emission λ_max_ at ≈930 nm. Evaluating photostability, the BPN-BBTD nanoparticles exhibited minimal fluorescence loss after continuous 1 h irradiation with a 793 nm laser of 1 W cm^−2^ power density, underscoring the robust photostability. Assessing cytotoxicity on colon cancer cells (CT-26) revealed no evident harm at nanoparticle amounts up to 100 µg mL^−1^ (Figure 4) [43,44].

#### 2.1.5. Donor–Acceptor–Donor Dyes

An alternative potential NIR-II fluorescent material is the donor–acceptor–donor (D-A-D) dye, which exhibits controlled emission of light in the NIR-II range. A well-known example is CH1055, a standard D-A-D dye settled by Sun et al. [45]. Due to their extended conjugated backbones, organic NIR-II fluorophores often exhibit strong intermolecular interactions, making their excited states susceptible to quenching and attack. This issue has been credited to reaction with an aqueous medium, contributing to the low QY of dyes like CH1055. A popular remedy involves crafting S-D-A-D-S variants, incorporating protecting units on both sides of the D-A-D dyes.

Li et al. introduced the highly effective nanoprobe p-FE, built on the S-D-A-D-S (FE) dye architecture. In this design, 3,4-ethylenedioxythiophene (EDOT) serves as the donating unit, benzo[1,2-c:4,5-c′]bis([1,2,5]thiadiazol) (BBTD) as the accepting unit, and dialkylfluorene acts as the shielding element. The capability of EDOT to induce backbone twisting mitigates the delocalization of the lowest vacant molecular orbital. Encapsulation of FE within an aquaphobic core, poly(styrene-co-chloromethyl styrene)-graft-poly(ethylene glycol) (PS-g-PEG), yields the final p-FE probe. The calculated quantum efficiency of p-FE reaches up to 1.65%. Utilizing p-FE and SWCNTs enabled successful two-color fluorescence imaging of vasculature and tumors in vivo [46].

**Figure 5:**
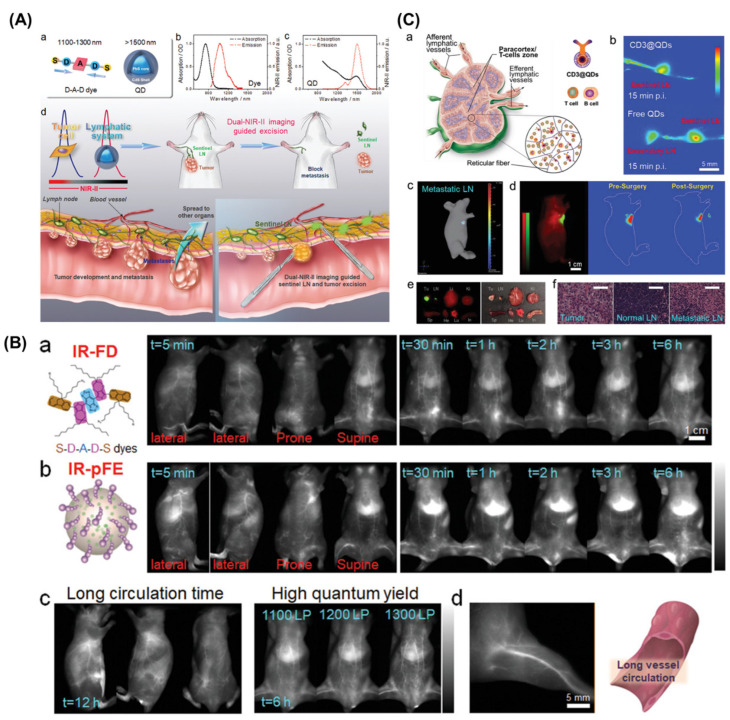
(**A**) NIR-II IGS of the sentinel lymph node. (**a**–**c**) NIR-II fluorescent materials engineered with D-A-D dye at the NIR-IIa wavelength and PbS QDs at the NIR-IIb wavelength. (**d**) Schematic representation of NIR-II IGS of sentinel lymph node. The D-A-D dye was intravenously administrated, after injecting QDs within the tumor. (**B**) Prolonged circulation and superior QY by IR-FD dye: (**a**) real-time observation of circulation of IR-FD in vivo. (**b**) Comparison of in vivo circulation between IR-FD and previous IR-pFE under identical imaging conditions. (**c**) Extended circulation duration resulted in prolonged retention within blood vessels. The elevated QY facilitated lower doses, and longer sub-NIR-II FI, enhancing penetrability and reducing scattering/autofluorescence from the tissues. (**d**) The extended circulation time and increased fluorescence intensity also enabled rapid NIR-II FI at a wavelength of 1200 nm. (**C**) Utilization of NIR-IIb FI for IGS of sentinel lymph node via anti-CD3 antibody-conjugated QDs: (**a**) illustration of sample accumulation in the sentinel lymph node. (**b**) Specific T-cell targeting in the sentinel lymph nodes facilitated by sample conjugates. (**c**) Development of a metastatic lymph node tumor model using 4T1 cells. (**d**) Application of NIR-II FI for IGS of sentinel lymph node by using a model having orthotopic 4T1 breast cancer. (**e**) Analysis of the biodistribution of dye and QDs signal. (**f**) Histological examination with H&E staining to differentiate healthy and metastatic lymph nodes [49] (Reproduced with permission from Copyrights 2020, WILEY).

In 2020, Tian et al. began by screening a combination of two NIR probe sets. They discovered a specific dye called IR-FD, featuring a D-A-D configuration, and PbS QDs, suitable for imaging in the NIR-IIa and NIR-IIb windows, respectively. Afterward, they examined the shapes of IR-FD and QDs. Both exhibited extremely small dimensions, measuring under 20 nm in diameter. The emitted light spectra from these two substances were unique, differing significantly, and not crossing over. Following the appropriate surface treatment of QDs and the process of PEGylation for IR-FD, employing a double NIR-II FI method enables the utilization of IR-FD dye to illuminate the tumor part. On the other hand, QDs serve to visualize sentinel lymph nodes. IR-FD and QDs displayed remarkable stability when exposed to high-power 808 nm laser radiation for up to 60 min, addressing concerns about signal decay for accurate measurement. We then analyzed the NIR-II fluorescent characteristics of all IR-FD, QDs, and a blend of IR-FD/QDs across distinct wavelength ranges, reaffirming that the chosen probes did not overlap. To assess penetration depth, a 1% Intralipid solution was used as a tissue mimic and compared QDs with the clinically utilized ICG probe. Observing fluorescence visualization of blood capillaries containing fluorescent material solutions, it was noted that QDs penetrated up to 9 mm with an SBR of ≈1.26, whereas ICG at 5 mm exhibited an SBR of ≈1.07. This demonstrated a notable enhancement in penetrability and contrasting ability in the NIR-IIb range with QDs compared to ICG (Figure 5) [47,48].

### 2.2. Inorganic Probes

The fluorescent probes containing inorganic fluorophores are termed inorganic fluorescent probes. In this section, we are going to discuss the inorganic fluorescent probes used for NIR-II FI-guided tumor surgery.

#### 2.2.1. Carbon Nanotubes

Dai et al. reported the utilization of SWCNTs coated with PEG in pioneering studies involving intravital FI within the NIR-II range. In solution, hydrophobic SWCNTs generate NIR-II fluorescence as a result of van Hove transitions across band gaps. Through surface modification employing PEG-conjugated lipids, the fluorescence QY of SWCNTs is elevated, rendering them biocompatible for in vivo applications. Subsequent advancements have showcased the application of SWCNT-based agents in various domains, including principal component analysis (PCA), targeted tumor imaging, and the assessment of hemodynamics in the peripheral arteries of mice. Remarkably, transcranial FI of the brain of the mouse was attained with impressive depth (>2 mm) and fine resolution (<10 μm) by channeling the NIR-II fluorescence of SWCNTs in the 1300–1400 nm spectral range [50,51,52].

In 2019, Ceppi et al. conducted an experiment examining how SWCNT imaging might impact tumor detection. They developed a specialized mouse model for ovarian cancer surgery, aiming to see how NIR-II FI could affect postoperative survival. They hypothesized that using FI during surgery would lead to better tumor removal, reducing the remaining tumor and ultimately improving the survival of the animals compared to those undergoing traditional visible-guided surgery. The study involved mice undergoing standard visible-tumor removal surgery without guidance and another group receiving the same surgery but with real-time detection of fluorescent tissues to aid removal. Out of the initial mice, two could not participate due to an ineffective injection of the SWCNTs probe. Seventeen mice underwent IGS, while eighteen had nonguided surgery. Among the nonguided surgery group, seven did not survive 72 h post-surgery due to various complications like bleeding, paralytic ileus, or bowel damage. Similarly, in the IGS group, seven mice did not reach the 72 h mark due to similar complications. Twenty-one animals survived beyond 72 h, but six of them had visible remaining tumors post-surgery, which could not be removed due to severe complications. Consequently, a total of fifteen animals were considered for the final survival analysis. The study found that the surgical and operating times were significantly longer in the IGS group, with an additional average time of around 14.7 and 13.3 min, respectively [53,54].

#### 2.2.2. Quantum Dots

The potential of SWCNTs for different biological uses has been hindered by their diverse size distribution and low light emission efficiency, despite their initial successes. NIR-II quantum dots (QDs) such as PbS, Ag_2_S, Ag_2_Se, and InAs have emerged as prominent contenders, boasting the highest fluorescence QY reported thus far, reaching up to 30% [55]. Notably, Ag_2_S-QDs were the first to exhibit NIR-II emission and hold promise for clinical utilization due to their absence of harmful heavy metal ions. Modified Ag_2_S-QDs, featuring branched six-armed PEG, display boosted gathering within tumors through the increased absorptivity and retaining effect. Compared to traditional fluorophores, Ag_2_S-QDs excel in visualizing lymph nodes, tissue perfusion, and angiogenesis in both visible and NIR-I windows. These QDs also serve as versatile platforms for constructing triggerable NIR-II fluorescent probes [56,57,58].

**Figure 6:**
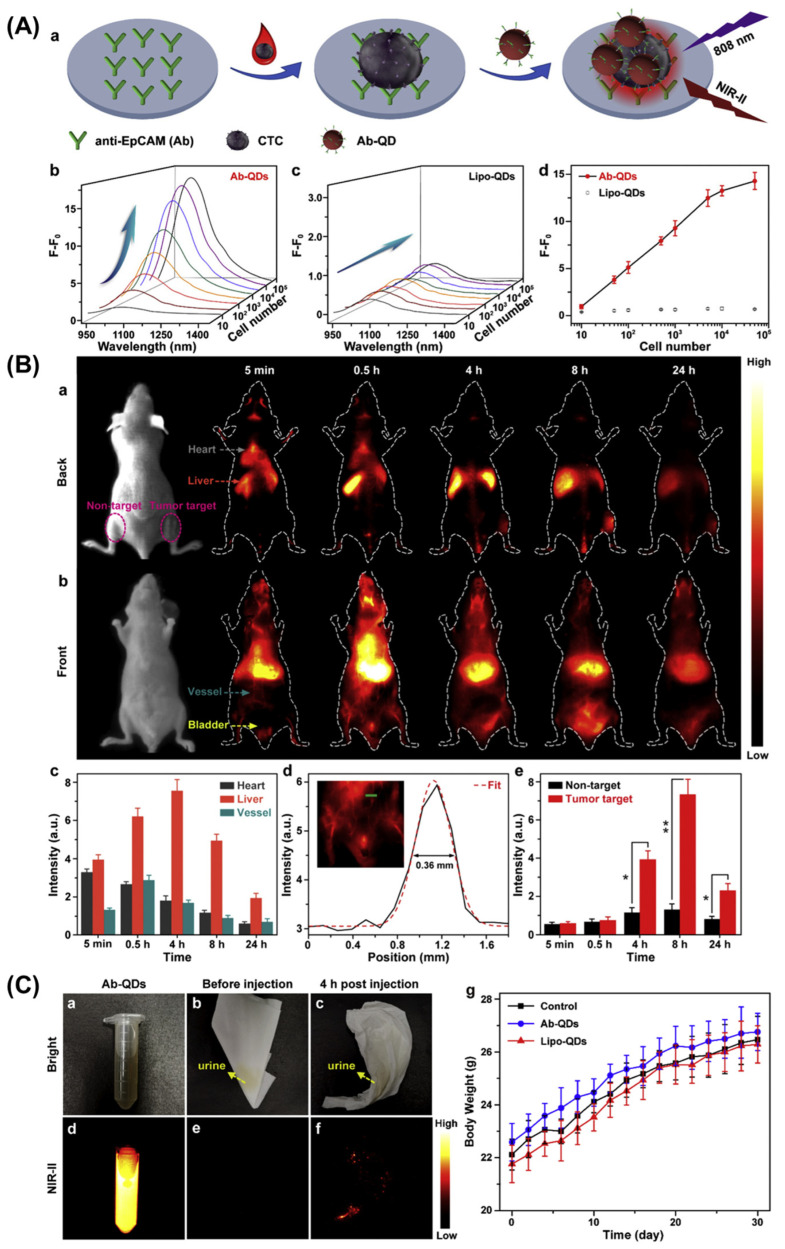
(**A**) (**a**) Diagrammatic representation of the process for detecting circulating tumor cells. Photoluminescence spectra of (**b**) antibody-conjugated quantum dots (Ab-QDs) and (**c**) liposome-encapsulated quantum dots (Lipo-QDs) for analyzing various quantities of MCF-7 cells. (**d**) Development of a calibration curve for the assay of MCF-7 cells. (**B**) Visible images and NIR-II fluorescence images were captured from the (**a**) dorsal and (**b**) ventral sides of nude mice having tumors following the injection of QDs at different time intervals. (**c**) NIR-II fluorescence intensity was observed in different body parts at various post-injection time points. (**d**) Typical cross-sectional NIR-II fluorescence intensity profiles. An inset displays a magnified view of the NIR-II fluorescence image depicting blood vessels. (**e**) NIR-II fluorescence intensity was recorded from the tumor and non-target regions, respectively. * *p* < 0.01, ** *p* < 0.005. (**C**) Images showing (**a**) sample dispersed in a PBS solution, (**b**) mice urine collected on paper before injecting sample, and (**c**) mice urine collected on paper 4 h after injection. NIR-II fluorescence images of (**d**) sample dispersed in a PBS solution, (**e**) mice urine before the injection of sample, and (**f**) mice urine 4 h after injection under irradiation at 808 nm. (**g**) Changes in the body weight of the mice in the absence of injection, and after intravenous administration of different samples, respectively [59] (Reproduced with permission from Copyrights 2020, ELSEVIER).

Lian et al., 2020, skillfully adjusted the Se/In ratio to produce CuInSe_2_ (CISe) QDs with an NIR emission wavelength adjustable between 920 nm and 1224 nm. Additionally, these CISe QDs exhibited an excitation band spanning from the UV to NIR region, which is highly desirable for a range of biomedical applications. By applying a thin ZnS shell coating, they achieved an impressive absolute NIR-II fluorescence QY of 21.8%, the highest reported for QDs free of Pb and cadmium Cd. Leveraging their strong NIR-II fluorescence, they showcased the utility of CISe nanoparticles for detection without autofluorescence for human breast cancer MCF-7 cells spiked in whole blood samples, with a detection limit as low as 12 cells per well in a 96-well plate. Furthermore, the CISe@ZnS nanoprobes were utilized for targeted tumor imaging in live mice, yielding an SBR of 5.8. These findings underscore the significant potential of the novel NIR-II fluorescent CuInSe_2_ nanoparticles in the realms of tumor diagnosis and IGS (Figure 6) [59].

Ongoing research endeavors have focused on enhancing the optical properties of Ag_2_S-QDs to unlock their therapeutic potential. For instance, a recent study by Zhao et al. introduced an innovative technique that effectively doubled the QY of Ag_2_S-QDs. By subjecting Ag_2_S-QDs dispersed in chloroform to femtosecond laser irradiation, a protective AgCl shell was formed, reducing structural defects and leading to an increase in fluorescence QY [60].

### 2.3. Multimodal NIR-II Fluorescence Imaging Probes

**Figure 7:**
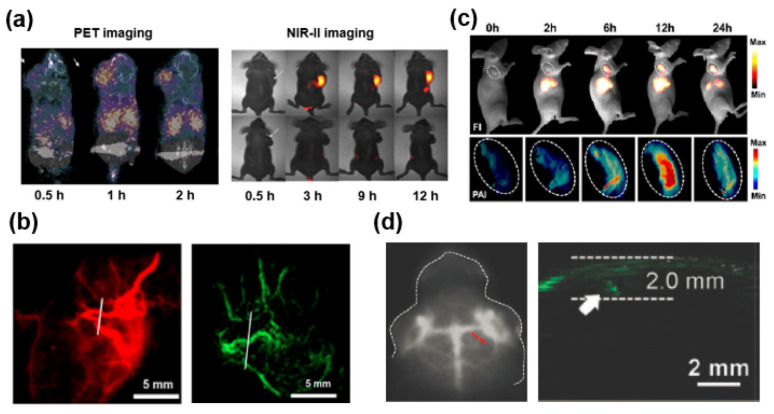
Utilization of multimodal FI probes incorporating NIR-II dyes including (**a**) PET and NIR-II FI of ^68^Ga-CHS_2_ [62]. Reused under Creative Commons Attribution License. (**b**) Imaging blood vessels through NIR-II and photoacoustic (PA) modalities using a sample [65] (Reproduced with permission from Copyrights 2017, AMERICAN CHEMICAL SOCIETY Publications). (**c**) Imaging SY1080 nanoparticles via NIR-II and PA techniques [66] (Reproduced with permission from Copyrights 2019, AMERICAN CHEMICAL SOCIETY Publications). (**d**) Visualizing orthotopic brain tumors with NIR-II and PA imaging employing TB1-RGD dots (red dash line; indicating the NIR-II fluorescence in brain tumor, white arrow; indicating photoacoustic differentiation of the brain tumor) [67] (Reproduced with permission from Copyrights 2018, WILEY).

Positron emission tomography (PET) imaging, renowned for its heightened sensitivity, has become a staple in cancer diagnostics within clinical settings [61]. Conversely, NIR-II imaging boasts advantages in accurately outlining tumor and resection areas. The pioneering work of Hong and Sun introduces the first instance of a NIR-II and PET multimodal fluorescent material, aiming for precise cancer detection. Leveraging the innovative base-catalyzed thiol-yne click chemistry, they easily synthesized the multimodal fluorescent material ^68^Ga-CHS_2_, which incorporates an NIR-II fluorescent dye, PET reporter, and selective polypeptide. By using ^68^Ga-CHS_2_, researchers can distinctly visualize tumor regions in mice through both NIR-II and PET imaging modes, yielding higher SBR, while the signal intensity notably decreases in blocking groups. Furthermore, NIR-II fluorescence IGS confirms thorough tumor dissection (Figure 7) [62].

Zhou et al. introduced NIR-II and magnetic resonance imaging (MRI) contrasting agents known as H-dots that are based on CH1055 and Mn-loaded dots. An in vivo investigation demonstrated that H-dots display higher uptake by the tumor, exhibiting minimal cytotoxic effects during the multimodal diagnostic imaging [63,64].

Typically, the pronounced absorption by NIR-II fluorescent material at or above 800 nm suggests its application for PTT and PAT [68]. Sun et al. synthesized a versatile and efficient material, designed based on the DD–A–DD skeleton called SYL. A dialkyl-substituted fluorene modality incorporated between the second donor diphenylamine and thiophene, the newly developed NIR-II fluorescent dyes, can effectively delineate tumor sites in both NIR-II FI and photoacoustic imaging modes [69]. Moreover, when subjected to 808 nm laser irradiation, SYL demonstrated an efficient photothermal effect, effectively inhibiting tumor growth. In another innovation by Sun et al., replacing a S atom with a heavier Se atom of the acceptor unit led to a bathochromic shift in fluorescence, yielding SY1080. Encapsulated in micelles, sample nanoparticles exhibited dual-mode imaging capabilities and PTT effects [66]. Additionally, Cen et al. developed TB1-RGD dots, which are linked with a targeting peptide covalently. These dots emitted at a NIR-II fluorescence of 1000 nm wavelength with a higher QY of 6.2%. Moreover, TB1-RGD dots selectively accumulated at tumor sites, emitting strong signals in both NIR-II and PA imaging modalities [70].

## 3. Future Prospects and Challenges

NIR-II FI has made significant strides in biomedical research and clinical applications, providing valuable insights into molecular and cellular processes. As technology advances, the exploration of NIR-III and higher wavelength fluorescence imaging opens up new frontiers with both promising prospects and inherent challenges [71].

NIR-III imaging offers longer wavelengths, enabling deeper tissue penetration. This enhances the potential for non-invasive imaging, particularly in organs located deep within the body, facilitating an earlier detection and monitoring of diseases. Higher wavelength imaging allows for improved spatial resolution and sensitivity. This can aid in the visualization of smaller structures at the molecular and cellular levels, leading to a more detailed understanding of biological processes [72].

NIR-III fluorescence can be integrated with other imaging modalities such as MRI and CT scans. This multimodal approach provides complementary information, offering a more comprehensive view of physiological and pathological conditions. NIR-III fluorophores often exhibit prolonged circulation times in vivo, resulting in an extended imaging window. This extended duration enhances the temporal resolution of imaging studies, allowing for real-time monitoring of dynamic biological processes [73].

The development of specific and efficient NIR-III fluorophores is a current challenge. Designing probes that possess both high QY and target specificity is crucial for the success of imaging applications. Ensuring the biocompatibility of NIR-III fluorophores is essential for their safe application in humans. Addressing concerns related to toxicity and potential side effects is a critical aspect of translating these technologies into clinical practice. Higher wavelength imaging requires specialized instrumentation. Overcoming the complexity of these systems, ensuring their accessibility, and simplifying the imaging process will be essential for widespread adoption in research and clinical settings. Establishing standardized protocols for NIR-III imaging and validating its reliability across different platforms are necessary for comparing results across studies and promoting the reproducibility of findings [74].

In conclusion, the future of NIR-III and higher wavelength fluorescence imaging holds immense promise for advancing our understanding of biological processes and improving clinical diagnostics. Overcoming current challenges will be pivotal in realizing the full potential of these technologies for transformative healthcare applications [75].

## 4. Summary and Outlook

NIR-II fluorescence IGS has demonstrated heightened efficacy with the use of NIR-II fluorescent probes owing to their advantageous features. These include superior penetration depth, surpassing that of visible and NIR-I probes, a crucial factor for effective imaging. Additionally, the high SBR, essential for precise tumor diagnosis within the body, contributes to their effectiveness. The high biocompatibility of the probe has facilitated in vivo experiments, while their biodegradability enhances safety and instills confidence for potential human trials. Their ultrasmall size enables quick diffusion, increasing availability throughout the entire tumor area and facilitating the easy crossing of the blood–brain barrier during brain tumor fluorescence imaging. Moreover, the targeting property makes NIR-II fluorescent probes promising agents for precise tumor targeting.

Furthermore, the application of NIR-II fluorescent probes in IGS has been found to significantly improve patient survival rates and decrease tumor recurrence. This technology has notably enhanced intraoperative accuracy in tumor margin differentiation. To further enhance the accuracy of NIR-II fluorescence imaging for IGS, strategies can include using tumor-targeting fluorescent probes for improved differentiation from normal body cells, employing activatable fluorescent probes to allow toggling between “ON” and “OFF” states as needed and utilizing probes that exhibit fluorescence in a larger wavelength range (NIR-III) to enhance penetration depth and signal-to-background ratio.

Despite the promising nature of NIR-II FI for IGS, its widespread application requires more robust evidence through human trials. Additionally, the practical implementation of NIR-III fluorescence imaging demands substantial efforts in laboratory research.
